# A Qualitative Modeling Approach for Whole Genome Prediction Using High-Throughput Toxicogenomics Data and Pathway-Based Validation

**DOI:** 10.3389/fphar.2018.01072

**Published:** 2018-10-02

**Authors:** Saad Haider, Michael B. Black, Bethany B. Parks, Briana Foley, Barbara A. Wetmore, Melvin E. Andersen, Rebecca A. Clewell, Kamel Mansouri, Patrick D. McMullen

**Affiliations:** ScitoVation, Research Triangle Park, NC, United States

**Keywords:** cellular mode-of-action, predictive toxicology, whole genome prediction, high-throughput toxicogenomics, pathway enrichment analysis

## Abstract

Efficient high-throughput transcriptomics (HTT) tools promise inexpensive, rapid assessment of possible biological consequences of human and environmental exposures to tens of thousands of chemicals in commerce. HTT systems have used relatively small sets of gene expression measurements coupled with mathematical prediction methods to estimate genome-wide gene expression and are often trained and validated using pharmaceutical compounds. It is unclear whether these training sets are suitable for general toxicity testing applications and the more diverse chemical space represented by commercial chemicals and environmental contaminants. In this work, we built predictive computational models that inferred whole genome transcriptional profiles from a smaller sample of surrogate genes. The model was trained and validated using a large scale toxicogenomics database with gene expression data from exposure to heterogeneous chemicals from a wide range of classes (the Open TG-GATEs data base). The method of predictor selection was designed to allow high fidelity gene prediction from any pre-existing gene expression data set, regardless of animal species or data measurement platform. Predictive qualitative models were developed with this TG-GATES data that contained gene expression data of human primary hepatocytes with over 941 samples covering 158 compounds. A sequential forward search-based greedy algorithm, combining different fitting approaches and machine learning techniques, was used to find an optimal set of surrogate genes that predicted differential expression changes of the remaining genome. We then used pathway enrichment of up-regulated and down-regulated genes to assess the ability of a limited gene set to determine relevant patterns of tissue response. In addition, we compared prediction performance using the surrogate genes found from our greedy algorithm (referred to as the SV2000) with the landmark genes provided by existing technologies such as L1000 (Genometry) and S1500 (Tox21), finding better predictive performance for the SV2000. The ability of these predictive algorithms to predict pathway level responses is a positive step toward incorporating mode of action (MOA) analysis into the high throughput prioritization and testing of the large number of chemicals in need of safety evaluation.

## Introduction

Gene expression changes have proven to be reasonable predictors of the dose-response for classical apical endpoints *in vivo*, i.e., the 2-year rodent bioassay (Ellinger-Ziegelbauer et al., 2008; Thomas et al., 2013). Toxicogenomic responses are also being used successfully to categorize developmental toxicants (van Dartel et al., 2010; Theunissen et al., 2011; Hermsen et al., 2012), and many approaches exist for evaluating similarities and differences in toxicogenomic responses across chemical groups (van Dartel et al., 2011). Different suites of genes that serve as transcriptional biomarkers of genotoxicity have been identified (Amundson et al., 2001; Iida et al., 2003; Dickinson et al., 2004; Ellinger-Ziegelbauer et al., 2008; Boehme et al., 2011; Li et al., 2015). Toxicology is now moving toward use of higher-throughput *in vitro* methods as a basis for screening compounds for subsequent testing and these screening analyses for transcriptomic changes are playing an increasingly prominent role in early stages of testing (Li et al., 2012; Hawliczek-Ignarski et al., 2017).

Even though the costs of full genome expression analysis technologies continue to fall, the large number of untested chemicals in commercial inventories have inspired the use of high-throughput transcriptomics (HTT) approaches for assessing gene expression changes. These technologies are based on the presence of a high degree of correlation between the expression of related genes across the genome (Eisen et al., 1998; Allocco et al., 2004; Fraser et al., 2004; Zhou and Gibson, 2004; Liang et al., 2018). Leveraging this interdependence, some HTT technologies measure the expression of relatively small subsets of “surrogate” genes and impute the balance of the genome using computational prediction models. The imputed equivalent to a whole transcriptome assay can then be used to make inferences about chemical targets using a variety of gene association techniques, followed by enrichment analyses to link gene expression profiles to known patterns of either cellular biology or of responses to chemical exposures.

One of the pioneering HTT efforts was Genometry’s L1000 platform^1^. The landmark genes used in the L1000 platform were derived using available public human gene expression data to determine genes with the most correlated expression changes across a range of cell types and chemical stressors, primarily from studies with pharmaceutical compounds. This correlation analysis yielded a set of 978 genes that were then used to computationally predict the remainder of the transcriptome (the inferred probes). The L1000 platform has been shown to be highly reproducible, and suitable for computational inference of expression levels of about 81% of non-measured transcript abundance (Subramanian et al., 2017). Results from the L1000 have been used successfully to predict molecular targets based on similarity analysis with responses to other pharmaceutical compounds (Subramanian et al., 2017). Because of the large number of chemicals in commerce or under development for commercial use that have little to no toxicity data, the promise for this type of approach in environmental science is substantial.

For toxicogenomic interpretation, L1000 data has been coupled with a novel chemical association algorithm using the Connectivity Mapping (CMAP) concept (Lamb et al., 2006). CMAP uses a large database of L1000 generated gene expression profiles derived from thousands of small molecule and genetic reagent exposures to multiple cell lines. Novel compounds can in turn be assayed on the L1000 platform and their measured gene expression used to search for non-random associations with expression signatures of tested compounds to infer commonality in function and cellular effects. While the CMAP concept was developed independently of the L1000 assay technology, the current public CMAP database has been derived from L1000 data due to the high throughput nature of the L1000 screening system^2^.

Existing full genome prediction models like the L1000 have primarily used pharmaceutical compounds as their test sets. The chemical space of commercial compounds is much larger than that of pharmaceutical compounds. It is not at all clear that any single predictive gene expression model will be equally effective across this broader landscape of chemical structures. This chemical diversity makes it difficult for the inference of specific modes of action for adverse effects. Highly adaptable or “tunable” modeling algorithms for predictive toxicogenomics that are computationally tractable and both time and cost effective would be more useful than any single, static platform.

In this study, we explored the application of gene expression prediction models to a more diverse chemical space, focusing on two primary goals. First, rather than using a fixed candidate gene set as predictors, we developed a more robust, data driven predictor selection. This process is intended to permit high fidelity gene prediction from any pre-existing gene expression data, regardless of species or platform used to generate the relative gene expression measurements. Such a data driven approach would allow for refinement of the predictor selection as new or additional data became available or could be tailored to particular exposure landscapes when existing predictors prove less than optimal. Our second goal was to use data-driven predictors set to computationally infer whole genome equivalent transcriptomic expression and then process those expression estimates qualitatively to elucidate conventional ontology enrichment results for inferring toxicogenomic mode of action (MOA). The robust data-driven predictor selection, independent of a specific gene expression technology, combined with whole transcriptome expression modeling and qualitative selection of differentially expressed genes for ontology enrichment could prove a valuable open-source approach to HTT chemical screening.

## Methods

We developed a novel set of HTT genes based on a broader suite of chemistries than previously investigated. Toward this end, we developed a qualitative approach based on classification models predicting three classes of probes: up regulated, down regulated, and unchanged. The context of selecting qualitative model over quantitative has been provided in **Supplementary Material**. This approach uses machine learning to select a set of surrogate genes using a publicly available toxicogenomics database containing gene expression changes resulting from exposure to a wide range of heterogeneous chemicals. Data resources, such as the TG-GATEs database have greatly expanded the chemical domain of transcriptomic data. The available TG-GATEs data was randomly split into a training set (75%) used to select the surrogate genes and fit the predictive model, and a test set (25%) used for model validation. The predictive performance of the resulting model was assessed based on pathway enrichment analysis comparing how major pathways were enriched using up-regulated and down-regulated genes for both the actual and predicted expression patterns. In a second step, we used the Genometry L1000 and another well-established Affymetrix whole genome toxicogenomics platform to gauge performance characteristics of the pathway enrichment approach.

### Data

For modeling purposes, we used primary human hepatocyte exposure data for 158 compounds in the Open TG-GATEs (Igarashi et al., 2015) database. Gene expression for the primary human hepatocyte exposures were run on Affymetrix HG-U133_Plus_2 microarrays, typically at three exposures (low, middle, and high), the actual values of which varied depending on the specific compound. Each exposure series used its own vehicle controls. Data were also typically sampled at 3-time points for most compounds: 2, 8, and 24 h. Gene expression in response to the 158 compounds across concentration and exposure times gave a total of 941 experimental conditions. The full listing of CEL files and samples available in Open TG-GATEs is listed in **Supplementary Material (Data Sheet 2)**.

### Selection of Surrogate Genes

**FIGURE 1:**
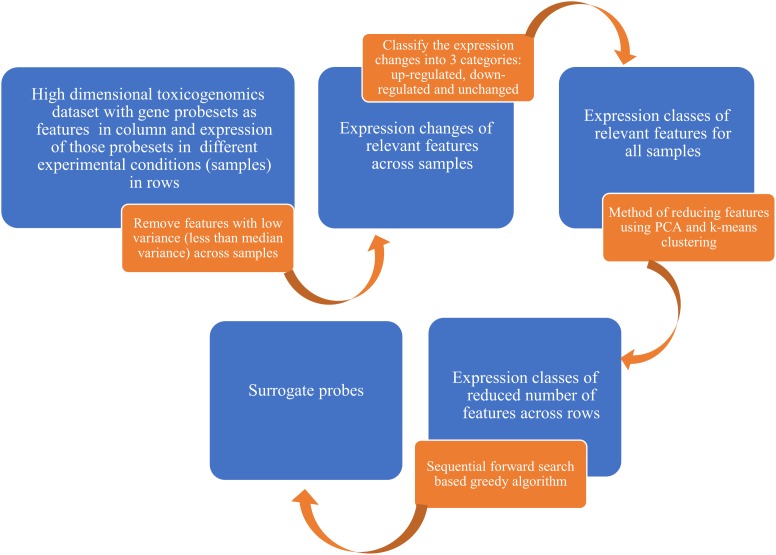
A flowchart for selecting a set of surrogate genes from largescale toxicogenomics data involving diverse training set. We started with high dimensional toxicogenomics data with gene probesets as features and exposure of those probesets in different experimental conditions as samples. After several filtering and feature selection process, we used a sequential forward search-based greedy algorithm to select the surrogate probes. See **Supplemental Figure S1** for the detailed algorithm.

Seventy-five percent of the total samples in the TG-GATEs were used for the selection of surrogate genes while the remaining 25% samples were used subsequently to validate the performance of the predictive qualitative models which used the new surrogate genes as predictors. The method of selecting a set of surrogate genes by using TG-GATEs database involved several steps and machine learning techniques (**Figure 1** and **Supplemental Figure S1**).

### Removal of Low-Impact Genes

Genes with a very low variance of expression across different cell lines and different experimental conditions contained very limited information. To minimize the computational burden, we removed the genes which had low variance across samples from downstream analysis. The criteria to remove low variance gene expressions depended on the distribution of expression from specific dataset. We then removed any gene with a variance lower than the median variance across samples. The TG-GATEs gene expression data were then categorized using thresholds of -0.1 for down-regulation and 0.1 for up-regulation. The threshold was selected because it provides a similar proportional distribution of up-regulated, down-regulated and unchanged genes across samples for each type of chemical in the heterogeneous and diverse pool of chemicals.

### Further Reduction of Features Using Unsupervised Clustering

A combination of principal component analysis (PCA) and k-means clustering was used to cluster the relevant (other than the very low variance) features into *k* small clusters. Representative features from each cluster were used to create a set of features that serve as inputs to a greedy algorithm (GA) to select the surrogate genes. The reduced set has *k* features (see **Supplemental Figure S1**). The optimum value for *k* for the k-means clustering was found using the Elbow method (Ketchen and Shook, 1996). The Elbow method computes the distortions using incremental cluster numbers. Here, to reduce computational complexity we set the increment as 500.

### Machine Learning Methods for Selection of Surrogate Genes

**FIGURE 2:**
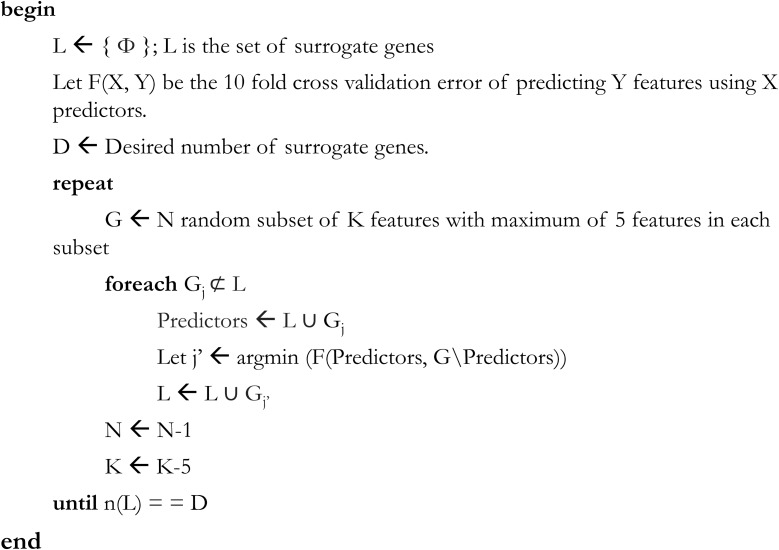
Algorithm for sequential forward search-based greedy algorithm to build set of surrogate genes. A 10-fold cross validation error of predicting Y features using X predictions was used as an objective function in each step of the greedy algorithm. The algorithm keeps building the set of surrogate genes in increment of five genes at a time until a desired number of surrogate genes are selected.

A sequential forward search-based GA was used to select the list of surrogate genes (see **Figure 2**). For this, we first identified co-regulated genes that have a similar direction (but not necessarily magnitude) of response irrespective of chemical treatment. Ultimately, the genes were curated to define a subset of genes that reliably represented the full genome. The GA was coupled to three different classification modeling algorithms consisting of support vector machine (SVM) (Cristianini and Shawe-Taylor, 2000), random forest (RF) (Breiman, 2001), and artificial neural network (ANN) (Ballabio et al., 2009). Each one of these coupled methods (GA-SVM, GA-RF, and GA-ANN) was performed separately leading to three sets of selected genes. A combined set of surrogate genes was created by taking those genes that appeared in at least two out of three different coupled methods.

Only qualitative models (up, down, and unchanged) were used throughout the selection of surrogate genes and the evaluation of their performances. The performance of each the three sets of surrogate genes was evaluated by training a model on 30% of the samples used in GA and validated on 25% of the holdout samples (validation set). SVM, RF, and ANN methods were used to validate the performance of surrogate genes found from GA-SVM, GA-RF, and GA-ANN respectively.

The performance of the combined set of the surrogate genes was evaluated using a consensus of all three methods (SVM, RF, and ANN). Prediction of the validation set was made using the combined surrogate genes with all three qualitative models and decision on final prediction was made on majority agreement of these models. When the three models predicted three different classes, the prediction was marked as “unchanged.”

### Pathway Enrichment Analysis

Pathway enrichment analysis was used to validate predictive ability of each set of surrogate genes. Cellular responses to chemical stimulus are achieved via concerted activation of biological pathways. Various efforts to map these pathways and the suites of genes associated with particular pathways have provided the scientific community with publicly available ontology databases. Here we used these publicly available ontologies to explore whether the incorporation of biological pathway information into the gene set analysis would improve prediction of whole genome response from the HTT gene subsets. We used a visualization technique we have employed previously (Clewell et al., 2014; Deisenroth et al., 2014; McMullen et al., 2014; Black et al., 2015; Andersen et al., 2017a,b) to perform traditional hypergeometric over-representation analysis for genes identified by our models as up- or down-regulated. Reactome is a curated biochemical pathway-based cell biology ontology with descriptions that progress from broad, collective functional categories (e.g., “metabolism” or “cell signaling”) to more defined sub-collections of functionally related cellular pathways, and finally to discrete biochemical cellular process pathways^3^. Pathways enriched in a toxicogenomics experiment can be summarized using a directed acyclic graph that captures these relationships between pathways in the ontology. Intensity of the color of nodes to indicate relative significance of their enrichment, and node size captures the relative number of elements from the query gene set found among that category’s elements. Together, the enrichment analysis and subsequent visualization provided both a statistically rigorous and intuitive snapshot of the processes perturbed by the compound.

We next applied a Pathway Similarity Index (PSI) to compare performance in terms of pathway enrichment analysis. The PSI has the following criteria:

• Pathway Similarity Index (PSI) finds similarity between actual and predicted pathways using up and down regulated genes of actual and predicted data respectively.
• PSI has a numeric value ranging from 0 to 1 where the highest value represents a perfect correspondence.
• Number of common pathways and number of query elements in each common pathway determines the numeric value of PSI.
• Finding larger set of common pathway element has a higher influence on PSI value than finding the smaller set.

The details of calculation of the PSI were as follows. Let *N_c_* be the number of pathways common between actual and predicted pathway enrichment, *N* be the number of pathways in the actual enrichment. Then PSI is calculated in equation 1 as follows:

(1) $$PSI=mean\left(\frac{N_{c}}{N},\partial \right)$$

Where ∂ is calculated in equation 2 as follows:

(2) $$\partial =\frac{\Sigma_{i=1}^{N_{c}}Q_{ci}}{\Sigma_{j=1}^{N}Q_{oj}}$$

Here, *Q_ci_* is the number of query element in *i*th common pathway; and *Q_oj_* is the number of query element in *j*th pathway in the actual enrichment.

## Results

### Generation of a Novel HTT Gene Set Using Machine Learning

Our approach removed features with variance lower than the median variance across samples for each gene to reduce computational complexity (see Methods, **Figure 1** and **Supplemental Figure S1**). This step reduced the number of probes from 54,675 to 27,338. The gene expression changes were then binned into three categories (up-regulated, down-regulated and unchanged) using a threshold of -0.1 for down-regulation and 0.1 for up-regulation.

These 27,338 probes were further reduced in the next step that involved unsupervised clustering with combination of PCA and k-means algorithm. The optimum number of clusters for the k-means clustering was found to be 10,000 using Elbow method (Ketchen and Shook, 1996). The optimum *k* found here captures 75% of the total variance. A representative probe was selected (nearest to the center of each cluster) to get the desired reduction from 27,338 to 10,000. After that, the GA based on each of the three classification techniques provided a predetermined number (2,000) of surrogate genes from these 10,000. Each of these gene sets contained 2,000 genes that should reliably predict the broader transcriptomic profiles irrespective of chemical treatment. A combined set was created (referred to here as the SV2000 surrogate set consisting of 2,332 probes) that contained genes which were present in at least 2 out of 3 surrogate sets.

### Validation of Prediction Performance of Surrogate Genes Using Pathway Enrichment Analysis

Differences between expression changes of individual genes measured across different transcriptomic platforms can be reconciled by assuming a pathway approach (Guo et al., 2006). Here, we tested whether this concept extends to differences between HTT and traditional transcriptomics experiments.

**Table 1** shows the comparison of prediction performance of expression classes in response to 100 μM 2,4-dinitrophenol using surrogate genes found from our algorithm using 3 different versions of GA (GA-SVM, GA-RF, and GA-ANN). In all the 3 versions, the algorithm was stopped after selecting 2,000 surrogate genes (see **Figure 2**).

**Table 1:** Comparison of prediction performances of expression classes in response to 100 μM 2,4-dinitrophenol using three different sets of surrogate genes found by 3 versions of GA.

Classification method in GA	Number of surrogate genes used	PSI
Random forest (GA-RF)	2,000	0.8132
Support vector machine (GA-SVM)	2,000	0.7552
Artificial neural network (GA-ANN)	2,000	0.7931


**The pathway similarity index (PSI) indicates the similarity between the pathways found using actual and predicted expression classes.**

**FIGURE 3:**
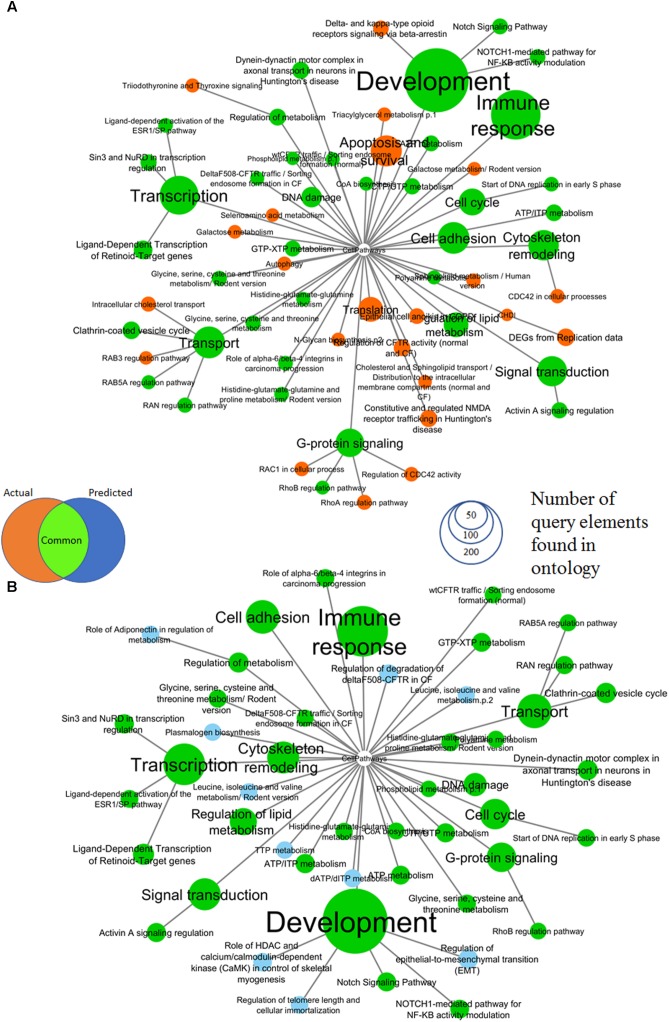
Comparison of MetaCore pathways enriched by up and down-regulated genes of **(A)** actual gene expression classes for TG-GATEs genes in response to 100 μM 2,4-dinitrophenol and **(B)** predicted gene expression using combined surrogate genes and a consensus prediction of all 3 prediction models. All colored nodes are significant at an enrichment FDR < 0.005 with a minimum of five query elements found in category elements. Ontologic enrichment of genes were performed against the public MetaCore Ontology and the enrichment was visualized. We have found that the predicted Affymetrix genes has a very similar enrichment profile as the actual gene expression. Thirty-eight (38) out of sixty-one (61) significantly enriched categories (shown in green) were common between actual and predicted gene expression classes.

The comparison of pathway enrichment using gene expression results from the full genome vs. predicted expression classes in response to 100 μM 2,4-dinitrophenol is shown in **Figure 3** where the combined set of surrogate genes (SV2000) was used. Prediction was made using a consensus of all three qualitative models (see Methods). Thirty-eight (38) out of sixty-one (61) significantly enriched pathways were found to be common with a PSI equal to 0.86. Significantly enriched pathways with most number of query element found in ontology include development, immune response, cytoskeleton remodeling, cell cycle, transport and transcription. Pathways common between the network determined with whole genome expression data and predicted network are in green.

**FIGURE 4:**
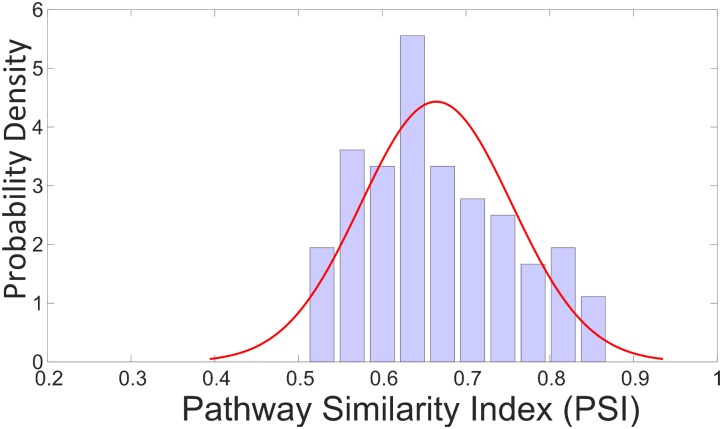
Probability distribution of PSI values for 100 random samples of validation set. The 100 samples are selected from the 25% holdout samples of TG-GATEs (validation set) keeping the distribution of categories same as original. The average of these PSI values was 0.64 with most of the values located between 0.6 and 0.8 and none of the values below 0.5.

PSI values for a total of 100 random samples from validation set (25% holdout samples from TG-GATEs), which has the similar distribution of use categories as the actual validation samples, were calculated and a distribution of these PSI values appears in **Figure 4**. The average of these PSI values was 0.64 with most of the values located between 0.6 and 0.8 and none of the values below 0.5.

To verify that the resulting PSI values are not obtained by chance, we did a Y-scrambling test where we randomly scrambled the samples in testing data to predict the expressions from the model created by non-scrambled training data. The average of these PSI values was 0.41 with this Y-scramble test which suggest that the result in our analysis was not obtained by chance.

**FIGURE 5:**
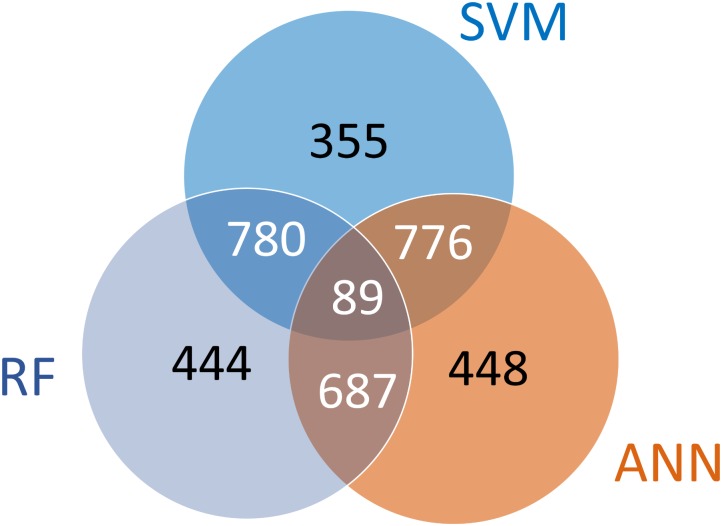
Patterns between the overlap of surrogate probes identified using 3 different machine learning approaches inside the greedy algorithm. Each of the sets contains 2,000 probes. The subsets labeled in white were used to combine the 3 sets of surrogate probes to create SV2000 which contains a total of 2,332 probes.

We next checked if there were patterns between the overlap of surrogate genes identified using different machine learning approaches. The diagram in **Figure 5** shows the number of common probes between the 3 selected sets. This number was 869 for SVM and RF, 776 between for RF and ANN, and 865 between the sets of SVM and ANN. A total of 89 probes were common to all three sets. The probes which were present in more than one set have a higher likelihood of serving as predictors of the remaining genome than the ones which are present in only one set. A total of 2,332 probes (SV2000 surrogate probes) were present in at least 2 sets and these probes were then used to predict the remaining genome. Interestingly, while the three machine learning approaches all produced predictive suites of surrogate genes, a large collection of genes (1,247, or 35%) were only identified by one algorithm. This behavior indicates that there is a degree of degeneracy of information in the transcriptome that HTT approaches build upon, i.e., the expression levels of many transcripts are approximately equally predictive.

## Discussion

### The Utility of Transcriptomics for Chemical Safety Evaluation

Gene expression microarrays and next-generation sequence technologies have been used to study functional changes from exposure to pharmacological, industrial, and agricultural compounds. However, a number of practical challenges have impeded the broader use of toxicogenomics for assessing hazards to human health, including the generally low throughput and expense of traditional microarray approaches. More limited, predictive transcriptomics gene sets should provide a viable alternative in leveraging the wealth of public gene expression data available to produce predictive models of transcriptomic change based on measurement of a small sub-sample of mRNA transcripts.

### HTT Approaches to Address Limitations of Conventional Transcriptomics

While HTT approaches are promising for evaluating gene expression changes, their utility for assessing response to environmental toxicants has not been fully evaluated. Our approach utilized TG-GATEs’ toxicogenomic data of human hepatocytes treated with diverse chemicals to select a set of surrogate genes. A sequential forward search-based GA has been used to develop three different sets of surrogate probes using three different classification approaches. A combined set was created (SV2000 surrogate set consisting of 2,332 probes) using the probes which are present in at least 2 out of 3 surrogate sets. This combined surrogate set was used to predict expression classes (up-regulated, down-regulated, and unchanged) of the remaining genome using a consensus prediction approach. Instead of directly comparing the expression levels, a pathway enrichment approach was used to validate the prediction performance.

### Comparison of Our Surrogate Genes With Existing Lists

In addition to the validation of our models, we used the pathway enrichment analysis approach and the measure of PSI to compare the performance of our selected set of genes with existing sets of surrogate genes developed by other methods. For this comparison, we chose the L1000 landmark genes and another list – the S1500 – that was designed to predict the whole transcriptome expression for toxicogenomic studies at the NIEHS National Toxicology Program. The genes in the S1500 list were based on a 5-step series of analyses to derive consensus gene sets that are highly correlated within a group of predictive genes, and which collectively represent known ontology associations of genes. The goal of the S1500 effort was to select gene sets with a high predictive capacity that are closely associated with defined ontology elements. To date, this approach has yielded 5,892 unique Affymetrix probes (HG_U133plus2 array based) representing 2,737 human genes that are collectively associated with 674 Reactome pathways^4^. All 978 landmark probes from Genometry’s L1000 platform are present among the 5,892 S1500 probes.

**FIGURE 6:**
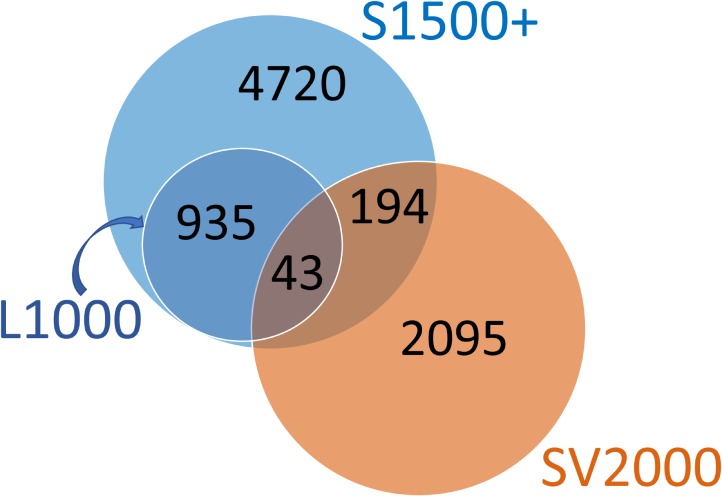
Overlap between S1500+ probe set, L1000 landmark probes and SV2000 surrogate probes. All 978 landmark probes from Genometry’s L1000 platform are present among the 5,892 S1500 probes; hence the L1000 set in the Venn diagram is pictured completely inside the S1500+ set. Among the 5,892 probes in S1500, 237 were found in the 2,332 SV2000 surrogate probes. For L1000, 43 of the 978 probes were present in SV2000 set.

We performed a probe-wise overlap analysis to understand the relationship between our identified SV2000 surrogate probes and existing sets (**Figure 6**). Among the 5,892 probes in S1500, 237 were found in the 2,332 SV2000 surrogate probes. For L1000, 43 of the 978 probes were present in our set. The small overlap between these different approaches is probably due to the redundancy within the gene expression data of the whole genome which is the conceptual basis behind the HTT approach: selecting a set of surrogate genes and predicting the remainder of the genome. These differences also appear to indicate that the information encoded in the gene expression of the three sets of selected genes is equally predictive despite differences in the identity of the surrogate probes.

To further investigate the predictivity of the three sets of genes, all 3 qualitative models (SVM, RF and ANN) were used to predict the remaining genome using SV2000, L1000, and S1500 surrogate probes. A decision on prediction was taken based on consensus of all three models. **Table 2** summarizes the results of this comparison showing that our set of surrogate genes (SV2000) provided somewhat higher PSI than the other surrogate sets. We interpret this result, i.e., that all three sets provide PSI values above 0.7, that there is no single set of surrogate genes that works in all cases and that the selection is likely to be technology and approach dependent. Our set of selected surrogate genes were powerful predictors when used within our fitted 3-methods (ANN-RF-SVM) consensus model and optimized for our MATLAB code. The code is available on GitHub^5^.

**Table 2:** Comparison of prediction performances of expression classes in response to 100 μM 2,4-dinitrophenol using three different sets of surrogate genes (our combined set, L1000 and S1500).

Predictor Set	Number of surrogate genes used	PSI
SV2000	2,332	0.8552
L1000	978	0.7097
S1500	5,892	0.7182


**The pathway similarity index (PSI) indicates the similarity between the pathways found using actual and predicted expression classes.**

### The Importance of HTT Data for Toxicology Applications

The use of an appropriate training set is crucial for meaningful interpretation of HTT data. For chemical safety and MOA applications, it is important to impute expression response from a diverse set of chemical compounds. Data included in both the CMAP and NIH’s LINCS collections is primarily derived from pharmaceutical compounds. These small molecule perturbations may differ substantially from those observed with industrial, consumer, or agricultural compounds and environmental toxicants or pollutants. Many pharmaceuticals have relatively discrete modes of actions and have one or a few possible targets in any given cell or tissue. In contrast, industrial compounds and agrichemicals often have multiple cellular targets and result in cellular perturbations involving many genomic pathways. Additionally, the biological information regarding modes of action with these compounds may be largely derived from experimental animals rather than humans. We include one example of this type of ontology enrichment in **Supplementary Material** for the agricultural fungicide fenbuconazole (used to primarily control molds on cereal crops) which has a liver MOA reportedly similar to that of phenobarbital, the common seizure-control barbiturate, which has a strong Cyp450 induction response in human liver.

To date, there have been few attempts to explore the application of similar gene expression prediction models to a more diverse chemical space or to correlate predictive expression analyses with existing *in vivo* dose response data relevant to industrial or agrichemical toxicity tests. One exception is a disease-centric approach to predict full equivalents for the Affymetrix HG-U133-Plus-2 array to fill in missing data from HG-U133a studies available in public repositories (Zhou et al., 2016). It remains to be seen how broadly applicable any single gene expression imputation method may be when applied to situations significantly displaced in chemical space or cellular response from those from which the data predictors were drawn. Additional characteristics of the training data sets, such as suitability of cell lines and representative tissues sampled in various studies, merely adds to the challenge in assessing any prediction platform for chemical toxicity screening.

### Continued Improvement of Predictive Transcriptomics Platforms

As technological developments decrease cost and increase throughput of full-genome transcriptomics, new toxicogenomics platforms are emerging alongside HTT. Novel sequence-based technologies that move beyond traditional next-generation approaches offer even higher throughput, such as BioSpyder’s TempO-Seq technology (House et al., 2017; Yeakley et al., 2017). Nonetheless, predictive transcriptomic modeling approaches will remain valuable tools as the National Toxicology Program implements their S1500 + initiative (NIOSH, 2013)^6^. Furthermore, HTT has applications in (1) efforts to align new data using emerging genomic technologies to legacy transcriptomics and (2) for data mining approaches of potentially useful gene signatures or more restricted gene sets for predicting the possibility of responses of human tissues exposed to various compounds.

The method introduced in this paper showed improved prediction performance compared to existing technologies. Our platform, though, also has some limitations that could be overcome in future versions. Identifying a set of an optimal surrogate genes from a virtually limitless domain of possible sets poses a technical challenge that generates computational constraints. A sequential forward search-based GA can become stuck in a local optimum and this can provide a false set of surrogate genes. Here, we mitigated this possibility by removing low-impact genes and using unsupervised clustering prior to the GA. Alternatively, the method might be improved by introducing computational measures to escape local minima. The prediction performance also depends heavily on selection of threshold for classifying the genes into three categories. Here, we have optimized these thresholds to 0.1 for up-regulation and -0.1 for down-regulation –a process that provided a similar proportional distribution of up-regulated, down-regulated and unchanged genes across samples for each type of chemical in the heterogeneous and diverse pool of chemicals. The future challenge can be using multiple toxicogenomics data and evaluate prediction performance across databases.

Any predictive transcriptomics technology that fails to keep pace with changes in respective species transcriptomic information will inevitably lose predictive power simply by ignoring emerging data that could be used for improving predictor selection and model training. For this reason, we plan to continually update our training sets to maximize the applicability domain of our models with the goal of increasing predictivity across a broader chemical space.

The other sets of surrogate genes used for comparison in this study are also likely to have specific strengths as well. A deep analysis of redundancy based on a large transcriptomic database could reveal the degree of overlap in information. Such study can be useful to extend and improve the set of surrogate genes. For example, a logical extension of the work presented in this paper would be to compare the power and concordance of chemical response prediction using our approach and the S1500 + predictive gene set as an independent assessment to the L1000 comparison presented here. This comparison could help clarify if there is some optimal predictive gene expression method, or some consistently highly predictive gene sets and ontology pathways that are more predictive of cellular changes associated with toxicity.

## Conclusion

In an attempt to select an optimal set of surrogate genes, we used a high-throughput toxicogenomics database, Open TG-GATEs, with expression of 54,675 probes in response to chemicals belonging to diverse classes. Given our emphasis on HTT for human risk assessment, we focused on human primary hepatocyte data in TG-GATEs to create a set of 2,332 surrogate probes (SV2000) to predict expression classes of the remaining genome. However, the data-driven predictor selection method presented here can be applied to any gene expression data, irrespective of species or platform used for data generation. This approach allows for refinement of the predictor selection as additional data become available.

Our process of generating SV2000 set made use of pathway enrichment of up-regulated and down-regulated genes as a measure of prediction performance – a strategy that eliminates difficulties in predicting changes of expression directly. Rather than having correlation coefficient or mean square error as prediction performance of direct expression prediction, we used a PSI that compared similarities and differences in the ontology pathways generated with up-regulated & down-regulated genes from both the actual and predicted gene expression classes. Our method is open source and showed significant improvement of prediction performance of the whole transcriptome compared to existing technologies for the cases examined to date. Together, these results highlight some of the challenges and opportunities of the emerging HTT approaches and their use in assessment of industrial and agrichemical compounds.

## Author Contributions

MB, BP, BF, SH, and BW generated and processed data for proof of concept. SH, MB, and PM processed data for the actual concept. MA, BW, and RC contributed in study, concept, and design. SH, MB, KM, and PM designed methods and algorithm. SH, MB, BW, KM, and PM wrote the manuscript. SH, MB, BP, BF, BW, MA, RC, KM, and PM reviewed and approved the manuscript.

## Conflict of Interest Statement

All authors were affiliated with and employed by ScitoVation, LLC. This manuscript is a product of ScitoVation funded by American Chemistry Council’s Long-Range Research Initiative. ScitoVation is not an academic institution. All authors declare no competing interest.

## Footnotes

1 http://genometry.com/

2 https://www.broadinstitute.org/connectivity-map-cmap

3 https://reactome.org/

4 https://ntp.niehs.nih.gov/go/S1500

5 https://github.com/ScitoVation/moa

6 https://ntp.niehs.nih.gov/iccvam/meetings/iccvam-forum-2016/7-niehs-tox21-508.pdf
