# Supplementary material for: A Qualitative Modeling Approach for Whole Genome Prediction Using High-Throughput Toxicogenomics Data and Pathway-Based Validation
Source: Front Pharmacol. 2018 Oct 2;9:1072. doi: 10.3389/fphar.2018.01072 (PMC6176017; doi:10.3389/fphar.2018.01072)
Supplement: Supplementary file 1 [file Data_Sheet_1.DOCX]

Supplementary Material

A qualitative modeling approach for whole genome prediction using high-throughput toxicogenomics data and pathway-based validation

**Saad Haider, Michael B. Black, Bethany Parks, Briana Foley, Barbara A. Wetmore, Melvin E. Andersen, Rebecca A. Clewell**^†^**, Kamel Mansouri, Patrick D. McMullen***

ScitoVation, Research Triangle Park, North Carolina, United States

^†^**Current address:** ToxStrategies, Inc., Cary, North Carolina, United States

***Correspondence:**

Patrick D. McMullen

[correspondingauthor@scitovation.com](mailto:correspondingauthor@scitovation.com)

Introduction

There are two fundamental components to an HTT approach. The first component of an HTT approach is to identify the set of surrogate genes. The second is the algorithm for extrapolating from the measured surrogate features to the balance of the transcriptome. In our original manuscript, we investigated whether using a machine learning approach with the publicly available large-scale TG-GATEs toxicogenomics database could inform selection of a novel HTT gene set that would support our qualitative pathway-based prediction approach.

In this supplementary document, we included detailed algorithm to select surrogate genes from TG-GATEs data. Later in this document, we included the context for selecting qualitative model instead of quantitative one. We used the L1000 gene set to demonstrate that a categorical approach in which the directionality of transcript changes is predicted rather than the magnitude can be useful for bridging different data sets. Furthermore, we showed that expression changes at the pathway level are more reliably extrapolated across platforms than changes at the individual transcript level.

Detailed algorithm to select surrogate genes from TG-GATEs data


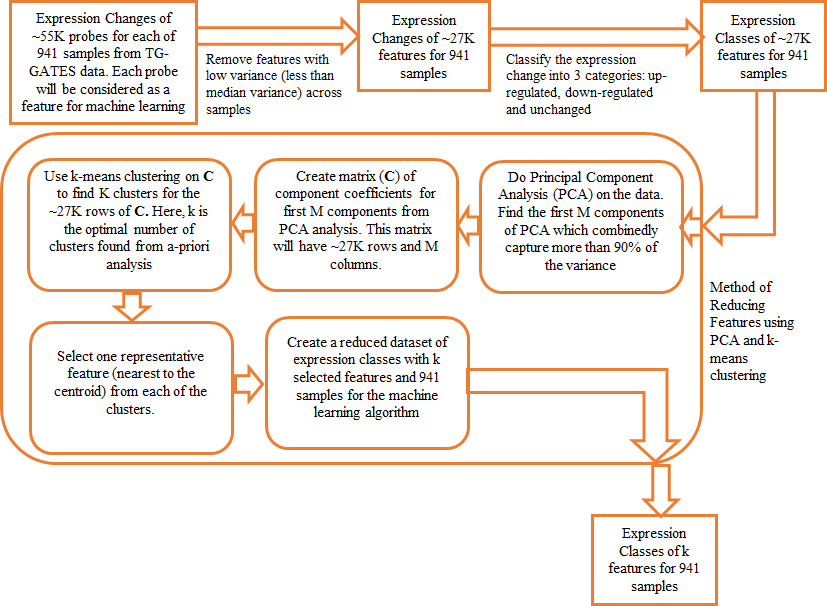


***Supplemental Figure S1a:*** *Detailed algorithm to select surrogate genes from TG-GATEs database. This part of the algorithm involves initial reduction of feature space by ignoring features with low variance across samples. It also involves further reduction of features using PCA and K-means clustering algorithm.*


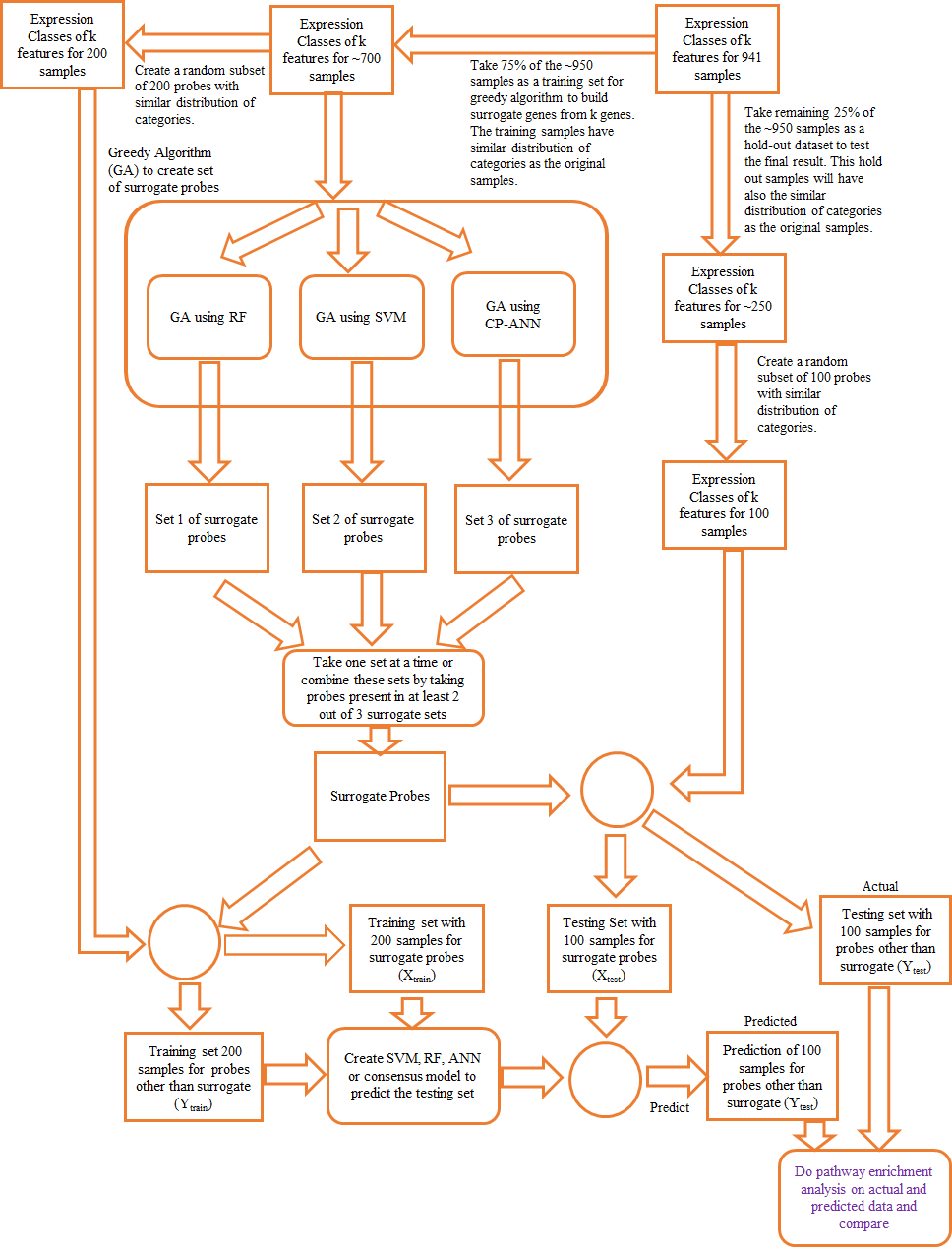


***Supplemental Figure S1b:*** *Detailed algorithm to select surrogate genes from TG-GATEs database. This second part involved sequential forward search based greedy algorithm to select the final set of surrogate genes. The surrogate genes were then used to train a model to predict expression of the remaining genome of a holdout set of samples from TG-GATEs. Finally, a pathway enrichment analysis was done on each of the actual and predicted hold-out expression classes.*

Challenges to predicting transcriptomics: relevance and data of predictor selection.

One aspect that confounds a direct comparison of dose response of current microarray technology and the L1000 inferred data is that the L1000 platform was designed to predict the equivalent of the Affymetrix U133a array probe set that has been superseded by newer platforms. This array design was released in 2002 and has been eclipsed in coverage by successive Affymetrix human whole genome coverage 3’ microarrays as well as those of other commercial vendors. Thus, it is now possible to measure expression of many thousands of human genes that were not part of the L1000 predicted probe set (see Supplemental Figure S2). Supplemental Figure S3 shows the overall ontology coverage of these 8,890 genes in the public GO Biological Process ontology and Reactome, showing that virtually all high-level parent ontology categories are represented by this set of genes.

*Supplemental Figure S2: Summary of annotated features represented on the human U133a equivalent array probe set, and the Affymetrix Titan HT_HGU133a_plus_PM array probe set. The 22,215 total non-control probes represented in the L1000 dataset correspond to 12,467 unique annotated genomic features (by Entrez Gene ID), while the Titan array probe set has a total of 54,613 non-control probes representing a total of 21,209 unique annotated genomic features. Of these, 8,890 Entrez gene IDs represented on the Titan array are not represented at all within the in the HG-U133a probe set.*

*Supplemental Figure S3: The GO-Biological Process and Reactome ontology categories represented by the 8,890 unique gene IDs present on the Affymetrix HT_HGU133_Plus_PM array that were absent in the earlier arrays.*

Methods for testing L1000 for the use of HTT for toxicity screening of environmental compounds

Data used

The 978 Genometry L1000 landmark genes were used as initial predictors to validate our qualitative approach. For model building and validation, we measured transcriptomic data in the Affymetrix GeneTitan and L1000 platforms in 3 cell lines (HepaRG, MCF7, and A673) after treatment with 3 agrichemicals (imazalil, fenbuconazole, and 2,4-dichlorophenonxyacetic acid) over 9 concentrations at 24-hour exposure.

In vitro transcriptomic studies

Test chemicals were chosen to represent well-studied agrichemicals with published in vivo and in vitro data. The three chemicals selected for *in vitro* exposures were; fenbuconazole (CAS # 114369-43-6) a triazole fungicide, imazalil (CAS # 35554-44-0), an azole pesticide, and 2,4-dichlorophenoxyacetic acid (CAS # 94-75-7), a chlorophenoxy herbicide. *In vitro* treatments used nine concentrations (0.001, 0.003, 0.01, 0.03, 0.1, 0.3, 1, 3, and 10 μM plus DMSO vehicle controls) and three cell lines (MCF7, A673, and HepaRG cells). Cells were exposed for 24h, and gene expression was measured using both Genometry L1000 ligation assay 384-well plates, and Affymetrix GeneTitan arrays (HT_HGU133_Plus_PM). The Affymetrix arrays used a common set of three vehicle controls per cell line experiment and three replicates for each exposure. The Genometry L1000 plates used 3 replicates per exposure, except for the HepaRG samples, which used 5 replicates per exposure due to high variation observed with this cell line during pilot exposures. Each plate had a total of at least 20 vehicle control wells that were used by Genometry for intra-plate normalization.

Cell culture and treatments

Cell culture methods and media were identical between the Genometry exposure experiments and the Affymetrix array experiments. HepaRG cells, HepaRG maintenance/metabolism medium supplement, HepaRG thaw/plate/general purpose medium supplement, HepaRG induction medium supplement, Williams E media (Gibco #A1217601), MEM (Cellgro #17-305-CV), DMEM (hyclone #SH30272.01), DMEM (cellgro #10-013-CV), 200mM Glutamax, fetal bovine serum, penicillin-streptomycin (pen/strep), sodium pyruvate, non-essential amino acids (NEAA), phosphate-buffered saline (PBS), and collagen I were purchased from Thermo Fisher Scientific (Waltham, MA). MCF7 cells and A673 cells were purchased from ATCC (Manassas, VA). 2,4-dichlorophenoxyacetic acid, fenbuconazole, and imazalil were purchased from Sigma-Aldrich (St. Louis, MO). RNeasy mini QIAcube kit was purchased from Qiagen (Valencia, CA). Vybrant MTT cell proliferation assay kit was purchased from Thermo Fisher Scientific (Waltham, MA).

HepaRG cells were thawed and plated according to the manufacturer’s protocol onto a collagen type 1 coated (2.5μg/well) 96-well plate (day 0) and allowed to attach for 24 h at 37^o^C in a humidified chamber with 95% relative humidity and 5% CO_2_. On days 1, 3, and 5, media was removed from the wells and replaced with 100μL/well of maintenance/metabolism supplemented media. On day 7, the media was removed and replaced with 100μL/well of induction supplemented media. On day 8, the cells were treated with chemical. MCF7 cells were cultured in MEM containing 10% FBS, 1X pen/strep, 2mM Glutamax, 1mM sodium pyruvate, and 1X NEAA and plated at 32,000 cells/well in a 96-well plate. A673 cells were cultured in DMEM (hyclone) containing 10% FBS and 1X pen/strep and plated at 26,000 cells/well in a 96-well plate. Following plating of the MCF7 and A673 cells, the cells were allowed to attach for 24 h before treatment. 3 biological replicates (independent experiments performed on independent batches of cells) were performed for all 3 cell lines.

DMSO stock solutions of 2,4-dichlorophenoxyacetic acid, fenbuconazole, and imazalil were added to culture media at a final DMSO concentration of 0.2%. The cells were exposed for 24 h to the DMSO vehicle (0.2%) or one of the following chemical concentrations: 0.001, 0.003, 0.01, 0.03, 0.1, 0.3, 1, 3, and 10μM.

Genometry L1000 assays

Chemical exposures were performed in 384-well plate format. Each plate contained at least 16-20 vehicle control wells. The 384-well plates were prepared in parallel batches where a batch consists of cells that were plated, treated and lysed at the same time. Plates were then shipped to Genometry for array scanning and expression measurement, and whole genome-equivalent (i.e., the entire U133a probe set) prediction. Data received from Genometry consisted of delimited text files with log_2_ transformed, intra-plate normalized expression values for the entire HT_U133a human array equivalent probe set.

Affymetrix gene expression analysis

Immediately following treatment, total RNA was collected from the treated cells using RNeasy kits according to the manufacturer’s protocol (Qiagen, Valencia, CA). NA samples were prepared for microarray hybridization using the GeneAtlas™ 3’ IVT Express Kit according to manufacturer’s protocol (Applied Biosystems, Carlsbad, CA). Briefly, total RNA was reverse transcribed to synthesize first-strand cDNA. The cDNA was then converted into a double-stranded DNA template for transcription. Amplified cRNA (aRNA) was transcribed *in vitro*, with incorporation of a biotin-conjugated nucleotide. The aRNA was then purified to remove unincorporated NTPs, salts, enzymes, and inorganic phosphate, and samples were fragmented to prepare the biotin-labeled aRNA samples for hybridization onto Affymetrix® GeneChip™. Samples were loaded onto a HT HG-U133+ PM GeneChip™ array plate and run on the GeneTitan® according to manufacturer’s protocol (Applied Biosystems, Carlsbad, CA). The GeneChip® HT HG-U133+ PM array plate analyzes the relative expression level of more than 47,000 transcripts and variants, including more than 38,500 well characterized genes and UniGenes, provides whole-genome coverage of the transcribed human genome on a single array in a 96-array configuration, and includes more than 54,000 probe sets and 1.3 million distinct oligonucleotide features. All gene array data discussed in this publication have been deposited in NCBI's Gene Expression Omnibus (Edgar et al., 2002) and are accessible through GEO Series accession numbers: GSE109508, GSE109509 and GSE109511.

Differential gene expression analyses

For both the Affymetrix array experiments and the L1000 array experiments, data were analyzed in R/BioConductor using the LIMMA and eBayes package on the normalized intensity values. Standard RMA log_2_ normalized expression was used for the Affymetrix array data analysis (Irizarry et al., 2003; Smyth, 2004; 2005).

Genometry L1000 plate data was received as intra-plate normalized, Log_2_ transformed intensity estimates. Each combination of cell line and chemical was extracted from these whole plate data and treated as discrete data sets. ANOVA subtraction was performed on each of the nine (3 cell lines by 3 chemicals) data sets to avoid any bias in estimated fold change related to differences in expression that could be attributed simply to fixed differences in either vehicle or treated samples between each of the replicates.

RMA normalized Affymetrix data and L1000 replicate corrected Log_2_ intensity values were subsequently analyzed identically, using LIMMA to fit a generalized linear dose response model to the expression data. Fitted models were used for empirical Bayesian tests of contrast for both overall dose-dependent response as well as individual concentration response relative to vehicle controls (9 contrasts using moderated *t* statistics). Benjamini-Hochberg FDR correction was applied to all statistical tests.

Preliminary Quantitative analysis

At the beginning of the quantitative analysis, continuous expression changes evaluated in the Affymetrix and L1000 platforms were directly compared in a quantitative manner. Spearman rank correlation was used to evaluate how the data from two platforms correlate.

We then derived an independent predictive model so we could directly compare gene expression estimates for the two technologies that were prepared by estimating gene expression from the same set of landmark genes. The goal was to evaluate the ability of the 978 landmark genes to predict data of one technology while training the model with data from the same chipset and from a different one. As L1000 is a proprietary technology, methods of L1000 prediction of Affymetrix data were not available. Hence, we applied a support vector machine (SVM) (Cristianini and Shawe-Taylor, 2000) regression model to evaluate predictivity of the 978 L1000 genes. For performance evaluation using training data from same technology, we have used 5-fold cross validation model for both Affymetrix and Genometry data where data of 978 genes from the corresponding technology has been used as predictors. For performance evaluation using training data from a different technology, we have used data of one technology to train an inference model (using data of that technology for 978 landmark genes as predictors) and predict the data of whole genome of another technology.

Genes which have very low variances across samples convey very little information in training a prediction model. To eliminate these low-impact genes in both Genometry and Affymetrix data, we have used probability distribution of variances: genes with expression changes lower than average of variances were not considered in either datasets (Supplemental Figure S4).


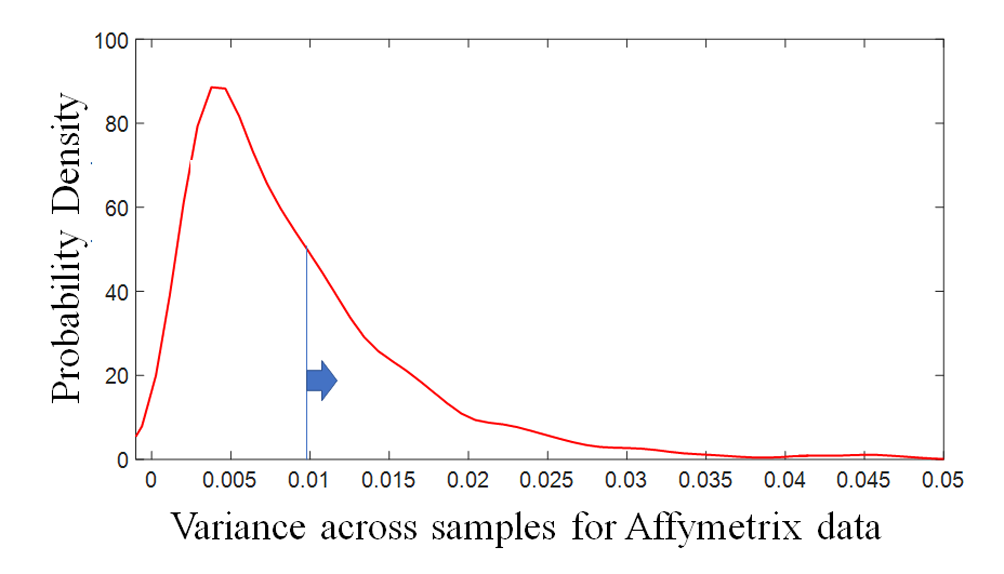


***Supplemental Figure S4:*** *Probability distribution of variance of genes across samples for Affymetrix data. Genes having variance greater than a threshold (mean of the variances) are selected for prediction.*

Spearman rank correlation was used to evaluate the prediction performance of the regression model. In addition to rank correlation, the performance of the SVM models was evaluated in fitting and cross-validation using the coefficients of determination R^2^ and Q^2^ (Todeschini et al., 2016), respectively. These two coefficients were calculated in equations 1 and 2 as follows:

$$R^{2}=1- \frac{\sum_{i=1}^{n_{TR}} \left( y_{i}-\hat{y}_{i} \right)^{2}}{\sum_{i=1}^{n_{TR}} \left( y_{i}-\bar{y} \right)^{2}}(1)$$

where $\hat{y}_{i}$ and $y_{i}$ are the estimated and observed responses of the *i_th_* element, respectively; $\bar{y}$ is the mean; and $n_{TR}$ is the number of training compounds.

$$Q^{2}=1-\frac{{\sum_{i=1}^{n_{EXT}} \left( y_{i}-\hat{y}_{i} \right)^{2}}/{n_{EXT}}}{{\sum_{i=1}^{n_{TR}} \left( y_{i}-\bar{y} \right)^{2}}/{n_{TR}}}(2)$$

where $n_{EXT}$ is number of test compounds and $n_{TR}$is the number of training compounds, $\hat{y}_{i}$ and $y_{i}$ are the estimated and observed responses, respectively, and $\bar{y}$ is the mean.

Qualitative analysis

We classified the datasets into 3 classes (up regulated, down regulated, and unchanged). The threshold of classification was found by maximizing the overlap between predicted Affymetrix (using Genometry data to train the model) and predicted Genometry (using Affymetrix data to train the model) classes.

Each of the Affymetrix and L1000 datasets was used to train the model to predict the expression classes of the other. Support vector machine (SVM) was used for the class prediction.

Balanced accuracy (BA) was used to evaluate the performance of classification models. Results for testing L1000 for the use of HTT for toxicity screening of environmental compounds

Qualitative approach to improve the prediction of the landmark genes

While HTT approaches promise to provide a cost-effective means of assessing compound effects, their equivalency to traditional toxicogenomics approaches (e.g., gene expression microarrays and next-generation sequencing) has not been well established. Here, we evaluated the predictivity of L1000 as a set of landmark genes by analyzing the consistency between the L1000 HTT and Affymetrix GeneTitan platforms with gene expression data for MCF7, A673, and HepaRG cells treated with 2,4-dichlorophenoxyacetic acid, fenbuconazole, and imazalil in 9-point concentration curves.

Continuous expression changes evaluated in the two platforms were not correlated (Supplemental Figure S5 and S6) and thus, cannot be directly compared in a quantitative manner. The quantitative estimates of expression change are substantially different in terms of “fold-change” determined through the two platforms. Technological differences between the L1000 and Affymetrix are a likely reason that one-to-one quantitative mapping between transcripts may be difficult. The correlation between the response of cells treated with different concentrations (Supplemental Figure S6) of the same compound was high for expression changes computed from Affymetrix measurements and L1000 measurements (average correlation 0.56 and 0.73 respectively). However, the correlation between corresponding samples between technology types was very low (-0.04).


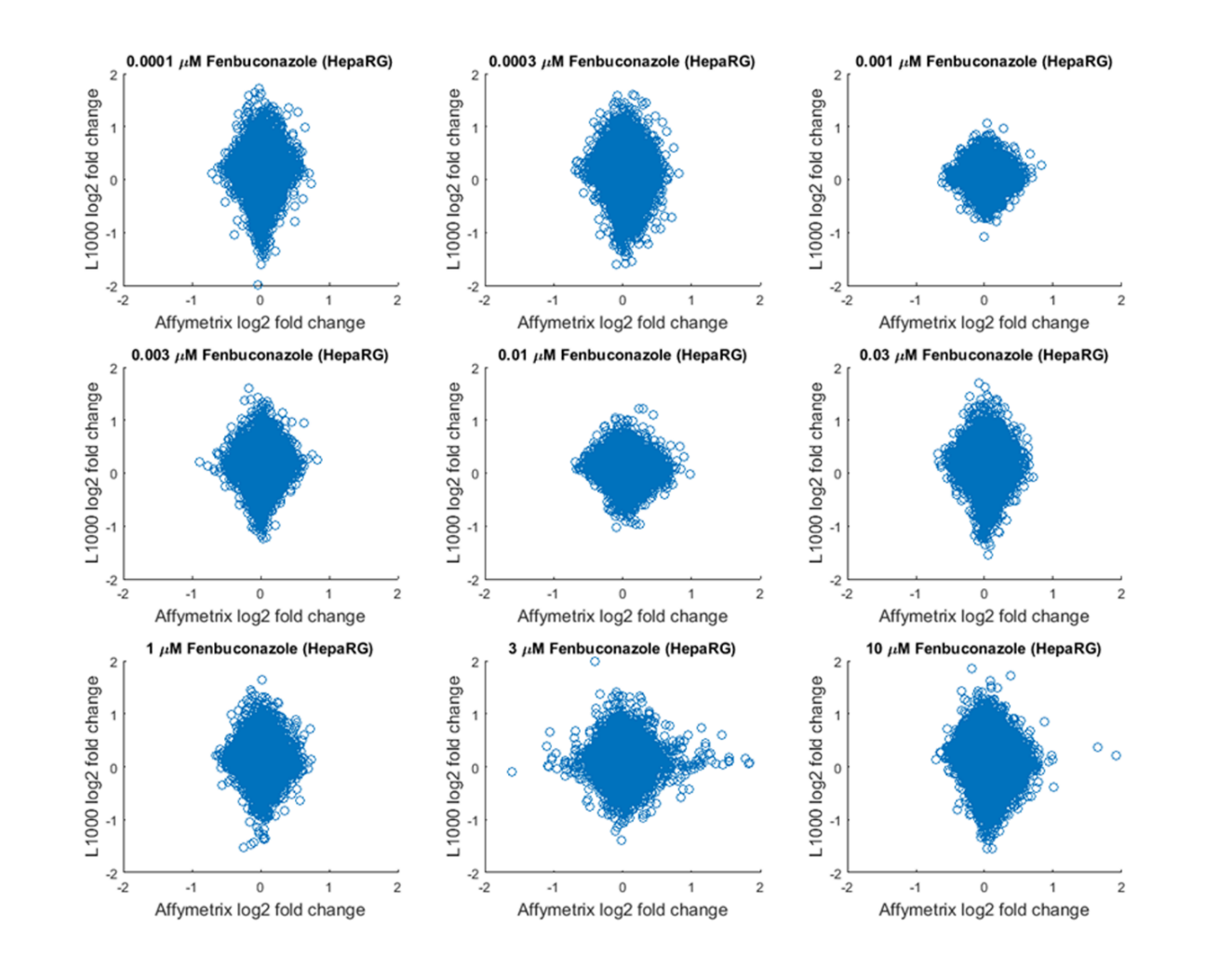


***Supplemental Figure S5:*** *Representative correlations between Affymetrix measurements and L1000 inference of gene expression. From top-left to bottom right, the concentration response of HepaRG cells to fenbuconazole. Correlations between the two technologies are essentially zero.*


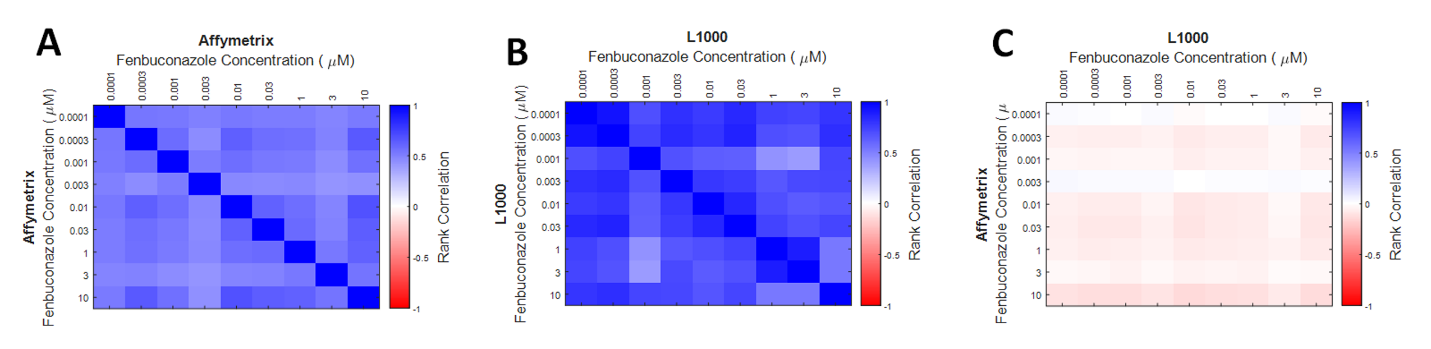


*Supplemental Figure S6: Probe-wise correlation of the response of HepaRG cells to various concentrations of fenbuconazole. Spearman rank correlation of Log_2_ fold change is indicated by color. On-diagonal values represent the correlation across a single concentration. (A) The response as measured by Affymetrix arrays was largely consistent across varying exposures of fenbuconazole (average pairwise correlation 0.56). (B) Similarly, the response inferred via the L1000 model is very self-consistent (average pairwise correlation 0.73). (C) The correspondence across technology platforms however, is largely nonexistent and in most cases, is actually anti-correlated (average pair-wise correlation -0.04).*

These differences between continuous expression changes across the two technologies might derive from fundamental differences in the technology platforms. Thus, rather than directly comparing the transcripts, as a second attempt to use the continuous data, we evaluated the predictivity of the L1000 genes by building regression models. To test this, we derived an independent predictive model to directly compare gene expression estimates for the two technologies by estimating gene expression from the same set of surrogate genes. The goal was to evaluate the ability of the L1000 978 landmark genes to predict data of one technology while training the model with data from the same chipset and from a different one.

***Supplemental Table 1.*** *Regression results of different approaches for Affymetrix and L1000 data using support vector machine. The results are based on 100 genes which were randomly selected.*

|  | **Regression Results** | | |
| --- | --- | --- | --- |
| Affymetrix | Cross Validation | Rank Correlation | 0.2975 |
|  |  | Q^2^ | 0.0361 |
|  | Trained on Genometry and Tested on Affymetrix | Rank Correlation | 0.0452 |
|  |  | R^2^ | -5.1549 |
|  | | | |
| Genometry | Cross Validation | Rank Correlation | 0.8467 |
|  |  | Q^2^ | 0.7082 |
|  | Trained on Affymetrix, Tested on Genometry | Rank Correlation | 0.0044 |
|  |  | R^2^ | -0.8984 |

The overlap between Affymetrix and Genometry dataset after the low-impact genes were removed has a total of ~13,000 genes. The SVM regression model was evaluated using 5-fold cross validation and train-test prediction (regression using SVM) results for Affymetrix and Genometry data for 100 randomly selected genes from the previously reduced set of ~13000 genes (Supplemental Table 1). For these 100 genes, the distribution of variances across samples were kept similar to the original set. The results are shown in terms of average rank correlation between actual and predicted expressions; it also shows coefficient of determination (average) R^2^ or Q^2^. The prediction of continuous measures of expression change showed poor correlations when we trained the model using data from one platform and tested it on data from another platform. For example, Affymetrix prediction using Genometry as training showed a rank correlation of 0.0452 and L1000 prediction using Affymetrix as training showed a rank correlation of 0.0044.

As one-to-one mapping between was unsuccessful across the two technologies, we converted the continuous transcriptomic data into discrete categories based on expression changes (see Methods). Genes were classified as up-regulated, down-regulated and unchanged. The goal was to see whether this qualitative strategy could play the role of a translator between the two technologies and allow data from the two platforms to be more readily comparable. The threshold for classification of these classes was set as described in the Methods.

The models for predicting classes instead of directly predicting expression changes of whole genome from 978 landmark genes from L1000 platform showed improved performance in terms of balanced accuracy of predicted classes for the same 100 genes randomly selected for regression (Supplemental Table 2). The BA for train-test prediction is better using the classification model than the rank correlation using the regression model. The Affymetrix class prediction using Genometry as training showed a BA of 28% while the Genometry class prediction using Affymetrix as training showed a BA of 25%. In each case, cross validation result shows better performances than train-test validation.

***Supplemental Table 2.*** *Classification results of different approaches for Affymetrix and L1000 data using support vector machine. The results are based on 100 genes which were randomly selected.*

|  | Classification Results | | |
| --- | --- | --- | --- |
| Affymetrix | Cross Validation | Balanced Accuracy | 55% |
|  | Trained on Genometry and Tested on Affymetrix | Balanced Accuracy | 28% |
|  | | | |
| Genometry | Cross Validation | Balanced Accuracy | 62% |
|  | Trained on Affymetrix, Tested on Genometry | Balanced Accuracy | 25% |

To visually compare the actual and predicted classes of these 100 genes in different samples, Supplemental Figure S7 was created using actual and predicted Affymetrix expression classes of those genes in response to 3 chemicals over 9 concentrations (27 samples) at 24-hour exposure of HepaRG cell line. The classification balanced accuracy (BA) for this data was 28%. While a 28% BA in classification model is low, it may be somewhat improved over a rank correlation of 0.0452 using the regression model, the poor concordance between the two gene sets is clear from the visual comparison.


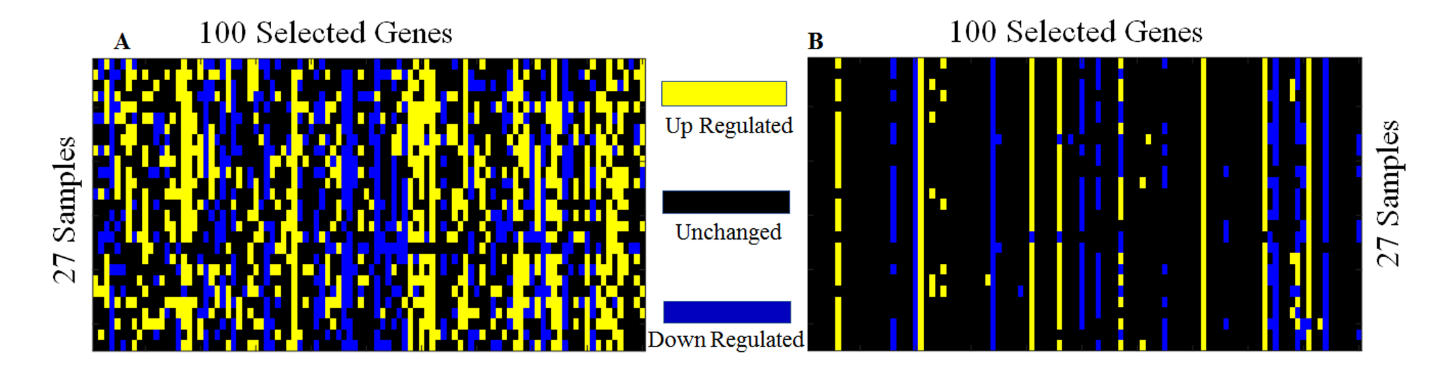


***Supplemental Figure S7:*** *Comparison of Affymetrix (A) actual gene expression for 100 previously selected genes in response to 3 chemicals over 9 concentrations (27 samples) at 24-hour exposure of HepaRG cell line and (B) predicted expression for the same samples from training the model by Genometry data. The classification BA is 28%.*

Pathway enrichment analysis for cross technology prediction

We used the L1000 gene set to demonstrate how reliably we can extrapolate expression changes at the pathway level across different technological platforms. A comparison of pathway enrichment was generated from up-regulated and down-regulated genes of actual and predicted Affymetrix gene expression classes for HepaRG cell line in response to 0.01 µM fenbuconazole (Supplemental Figure S8). Sixty three (63) pathways are significantly enriched by up-regulated and down-regulated genes of actual Affymetrix gene expression classes. Among these 63 pathways, 22 of them were reproduced by the predicted expression where Genometry data was used to train the predictive model. Significantly enriched pathways with most number of query element found in ontology include development, immune response, cell adhesion, translation, signal transdaction, cytoskeleton remodeling, and transcription. The pathway similarity index (PSI) between the was found to be 0.52.


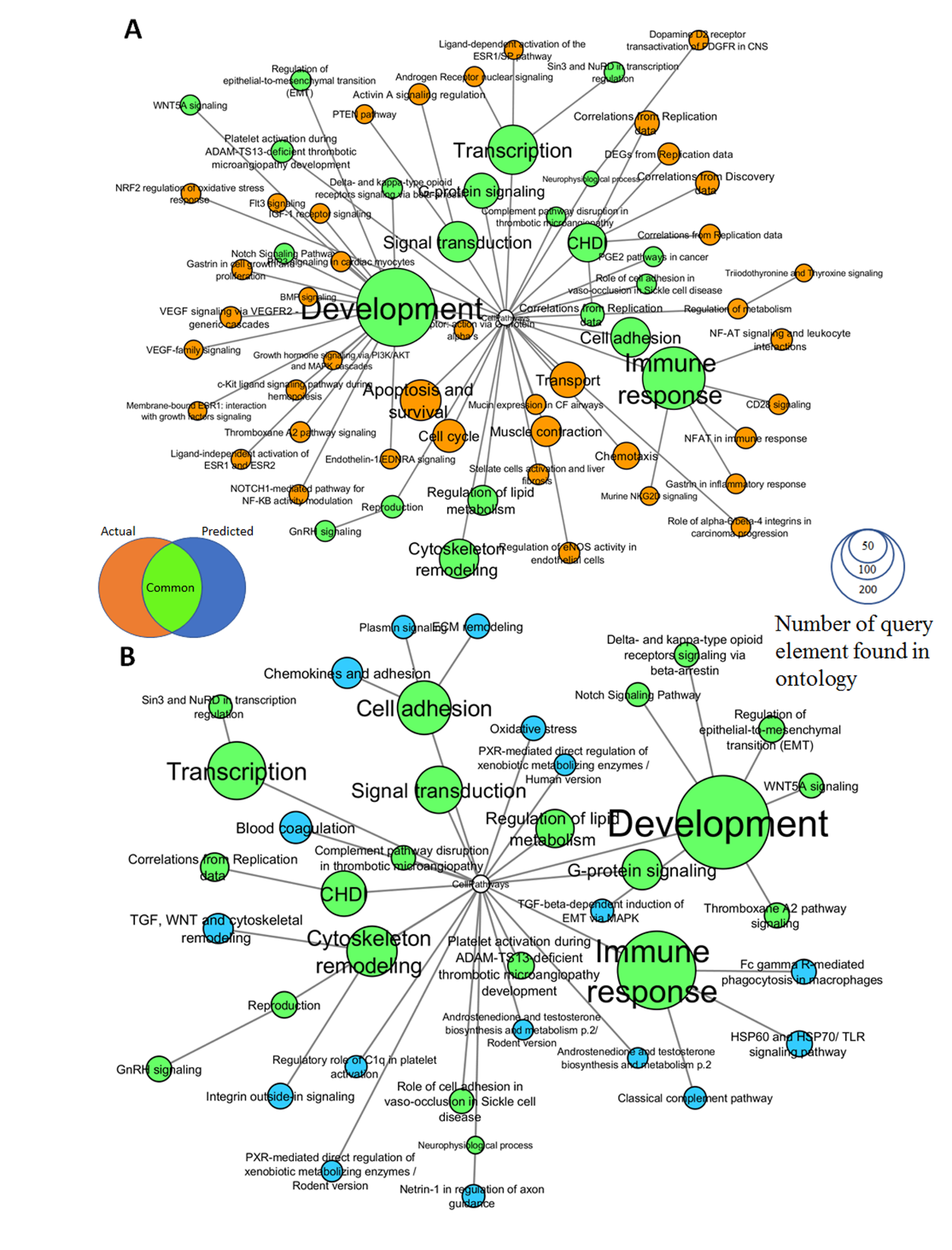


***Supplemental Figure S8:*** *Comparison of pathway enrichments generated from up & downregulated genes of (A) actual Affymetrix gene expression classes for HepaRG cell line in response to 0.01 µM fenbuconazole and (B) predicted Affymetrix gene expression classes where Genometry is used for training the model. All colored nodes are significant at an enrichment FDR<0.005 with a minimum of 5 query elements found in category elements. Ontologic enrichment of genes were performed against the public MetaCore Ontology and the enrichment was visualized. The predicted Affymetrix genes from Genometry provided a very similar enrichment profile as did the actual gene expression from the Affymetrix platform. Twenty-two (22) out of sixty-three (63) significantly enriched categories (shown in green) were common between actual and predicted gene expression classes (see the green nodes in the maps).*

Ontology enrichment of an example agrichemical (fenbuconazole) with well documented mode of action

The widely used crop fungicide, fenbuconazole is reported to have a phenobarbital-like Cyp450 induction response. Phenobarbital, a pharmaceutical agent used to control seizures, has a known primary cellular mode of action, targeting GABAA receptors in neural cells, and potentially blocking calcium ion pumps as well (ffrench-Mullen et al., 1993; Mathers et al., 2007). At high doses in rodents, phenobarbital activates the CAR-receptor leading to a series of cellular events that lead to hepatocyte proliferation, liver enlargement, and cancer on long-term exposures. These hepatic responses in rodents are of questionable relevance to humans (Elcombe et al., 2014). The Cyp450 induction seen with phenobarbital is related to its complex metabolism and relatively long half-life in the body (Waxman and Azaroff, 1992; Sergent et al., 2009; Zhu, 2010). Fenbuconazole, while also shows a broad suite of Cyp450 induction, has also been reported to cause increases in liver weights, centrilobular hepatocellular hypertrophy, hepatocellular vacuolation, cellular proliferation, alterations in triglyceride and cholesterol metabolism, and changes in cell cycle and mitotic functions (Waxman and Azaroff, 1992; Zhang et al., 2010; Zhu, 2010). Fenbuconazole is a demethylation inhibitor, and interrupts growth regulation in fungus infections of crops. While aspects of its cellular mode of action is similar to phenobarbital, its hepatocellular responses at the transcriptomic level are very broad, as indicated in our visual depiction of Reactome enrichment with differentially expressed genes (Supplemental Figure S9).

*Supplemental Figure S9: Reactome enrichment with up-regulated genes in HepaRG cells from the L1000 and Affymetrix experiments with exposure to Fenbuconazole. Genes used were statistically significant (FDR<0.05) for a dose dependent response across all concentrations and which were up-regulated by maximum observed fold change at any concentration. Nodes in red were enriched with genes from the L1000 data, those in green from the Affymetrix experiment and those in mustard were enriched with gene sets from both experiments.*

Comparison of probe-wise correlation of the responses of same chemical across different cell lines in Affymetrix data.

**

*Supplemental Figure S10: Comparison of probe-wise correlation of the responses of HepaRG, MCF7 and A673 cells to various concentrations of fenbuconazole. Spearman rank correlation of Log_2_ fold change is indicated by color. Although the concentration responses to fenbuconazole within same cell line are correlative, they do not correlate across different cell lines. This shows that the measured values in Affymetrix data were not mere noise.*

**

*Supplemental Figure S11: Comparison of probe-wise correlation of the responses of HepaRG, MCF7 and A673 cells to various concentrations of imazalil. Spearman rank correlation of Log_2_ fold change is indicated by color. Although the concentration responses to imazalil within same cell line are correlative, they do not correlate across different cell lines. This shows that the measured values in Affymetrix data were not mere noise.*

**

***Supplemental Figure S12:*** *Comparison of probe-wise correlation of the responses of HepaRG, MCF7 and A673 cells to various concentrations of 2,4-dichlorophenonxyacetic acid. Spearman rank correlation of Log_2_ fold change is indicated by color. Although the concentration responses to 2,4-dichlorophenonxyacetic acid within same cell line are correlative, they do not correlate across different cell lines. This shows that the measured values in Affymetrix data were not mere noise.*

# References

Cristianini, N., and Shawe-Taylor, J. (2000). *An introduction to support Vector Machines: and other kernel-based learning methods.* Cambridge University Press.

Elcombe, C.R., Peffer, R.C., Wolf, D.C., Bailey, J., Bars, R., Bell, D., et al. (2014). Mode of action and human relevance analysis for nuclear receptor-mediated liver toxicity: A case study with phenobarbital as a model constitutive androstane receptor (CAR) activator. *Crit Rev Toxicol* 44(1)**,** 64-82. doi: 10.3109/10408444.2013.835786.

ffrench-Mullen, J.M., Barker, J.L., and Rogawski, M.A. (1993). Calcium current block by (-)-pentobarbital, phenobarbital, and CHEB but not (+)-pentobarbital in acutely isolated hippocampal CA1 neurons: comparison with effects on GABA-activated Cl- current. *J Neurosci* 13(8)**,** 3211-3221.

Irizarry, R.A., Hobbs, B., Collin, F., Beazer-Barclay, Y.D., Antonellis, K.J., Scherf, U., et al. (2003). Exploration, normalization, and summaries of high density oligonucleotide array probe level data. *Biostatistics* 4(2)**,** 249-264. doi: 10.1093/biostatistics/4.2.249.

Mathers, D.A., Wan, X., and Puil, E. (2007). Barbiturate activation and modulation of GABA(A) receptors in neocortex. *Neuropharmacology* 52(4)**,** 1160-1168. doi: 10.1016/j.neuropharm.2006.12.004.

Sergent, T., Dupont, I., Jassogne, C., Ribonnet, L., van der Heiden, E., Scippo, M.L., et al. (2009). CYP1A1 induction and CYP3A4 inhibition by the fungicide imazalil in the human intestinal Caco-2 cells-comparison with other conazole pesticides. *Toxicol Lett* 184(3)**,** 159-168. doi: 10.1016/j.toxlet.2008.11.009.

Smyth, G.K. (2004). Linear models and empirical bayes methods for assessing differential expression in microarray experiments. *Stat Appl Genet Mol Biol* 3**,** Article3. doi: 10.2202/1544-6115.1027.

Smyth, G.K. (2005). "Limma: linear models for microarray data.," in *Bioinformatics and Computational Biology Solutions using R and Bioconductor,* ed. V.C. R. Gentleman, S. Dudoit, R. Irizarry, W. Huber (New York: Springer), 397-420.

Todeschini, R., Ballabio, D., and Grisoni, F. (2016). Beware of Unreliable Q2! A Comparative Study of Regression Metrics for Predictivity Assessment of QSAR Models. *Journal of Chemical Information and Modeling* 56(10)**,** 1905-1913. doi: 10.1021/acs.jcim.6b00277.

Waxman, D.J., and Azaroff, L. (1992). Phenobarbital induction of cytochrome P-450 gene expression. *Biochem J* 281 ( Pt 3)**,** 577-592.

Zhang, J.G., Ho, T., Callendrello, A.L., Crespi, C.L., and Stresser, D.M. (2010). A multi-endpoint evaluation of cytochrome P450 1A2, 2B6 and 3A4 induction response in human hepatocyte cultures after treatment with beta-naphthoflavone, phenobarbital and rifampicin. *Drug Metab Lett* 4(4)**,** 185-194.

Zhu, B.T. (2010). On the General Mechanism of Selective Induction of Cytochrome P450 Enzymes by Chemicals: Some Theoretical Considerations. *Expert opinion on drug metabolism & toxicology* 6(4)**,** 483-494. doi: 10.1517/17425250903578642.
